# A comparative analysis of health surveillance strategies for administrative video display terminal employees

**DOI:** 10.1186/s12938-019-0737-z

**Published:** 2019-12-11

**Authors:** Saki Gerassis, Alberto Abad, Javier Taboada, Ángeles Saavedra, Eduardo Giráldez

**Affiliations:** 1 Department of Natural Resources and Environmental Engineering, University of Vigo, Vigo, Spain; 2 Department of Statistics and Operational Research, University of Vigo, Vigo, Spain

**Keywords:** Data mining in healthcare, Occupational health surveillance, Health strategies, Clinical decision support systems, Video display terminals (VDTs), Health informatics

## Abstract

**Background:**

The objective of this study was to develop a strategy to optimize medical health surveillance protocols for administrative employees using video display terminals (VDTs). A total of 2453 medical examinations were analysed for VDT users in various sectors. From these data, using Bayesian statistics we inferred which factors were most relevant to medical diagnosis of the main disorders affecting VDT users. This information was used to build an influence diagram to evaluate the time and monetary costs associated with each diagnostic test and define an optimal protocol strategy based on occupational risks.

**Results:**

Musculoskeletal and ophthalmological diseases were identified as the most frequent disorders among VDT users. The Bayesian network inferred age, sleep quality, activity level, smoking and the consumption of alcohol as risk factors. The blood count was the most costly test (5.23 USD/employee) and the second most costly test in time terms (4 min/employee), yet is a diagnostic test that has little influence on the medical decision regarding an employee’s capacity to perform their job.

**Conclusions:**

Current occupational health surveillance protocols for VDT users may lead to expenditure that is 54% greater than necessary. For many employees and employers, failure to perform a wide range of medical tests for occupational health surveillance purposes is subjectively perceived as a threat to health. Awareness needs to be raised of the appropriate role of different health areas, so as to optimize diagnostic efficiency on the basis of greater flexibility.

## Background

Health monitoring of employees is a preventive measure that is regulated in many countries [1–3]. Regulation typically requires medical testing in accordance with the risk implied by particular posts. The aim is to ensure the employee’s capacity to do their job free of illness and to prevent occupational diseases from worsening [4, 5].

Clinical decisions regarding diagnostic tests are conditioned by job-related medical protocols as regulated in each country. These protocols are developed on the basis of the risks to which employees are exposed in their workplace [4, 6]. Traditionally, preventive medical examinations involve general medical tests that in many cases are not directly related to workplace risks.

A current focus of interest is jobs in which visual display terminal (VDTs) are handled [7], as nowadays, computers and tablets are used in most jobs worldwide [8]. Specific protocols and various studies [4, 9, 10] have indicated that health surveillance in relation to VDTs should focus on two fundamental issues: an ophthalmological examination [11, 12] to detect eyestrain, irritation, redness, blurred vision and double vision; and a musculoskeletal system examination [13, 14], to identify bone and muscle injuries, such as spinal column problems. However, medical examinations typically include many other diagnostic tests—such as blood and urine tests or audiometry—which do not necessarily detect occupational health problems. This happens because employees and employers assume that an examination that excludes general tests is not synonymous with quality. Therefore, medical staff and prevention services feel pressurized, based on cultural and commercial reasons [15], to perform general tests that lead to an inevitable diagnostic duplicity with general medical care. This results in health system inefficiency [16], first in terms of an economic cost due to the repetition of medical tests, and second, due to mismanaged medical staff time, with the resulting longer waiting lists for consultations with doctors and for diagnostic tests [17]. The outcome is a decrease in health care quality and patient dissatisfaction [18].

To develop a solution for this problem, for employees using VDTs we analysed 2453 cases of medical surveillance examinations (dating from 2010 to 2016) in order to define a personalized medical protocol for the main diseases affecting this group. Quantifying the time and monetary costs of each test enabled us to explore strategies to optimize the health surveillance process. Our ultimate aim is to advance the development of personalized medicine by developing a decision-making tool that would inform medical staff from the perspective of both employee health and resource management.

## Results

### Evaluated employees

**Fig. 1:**
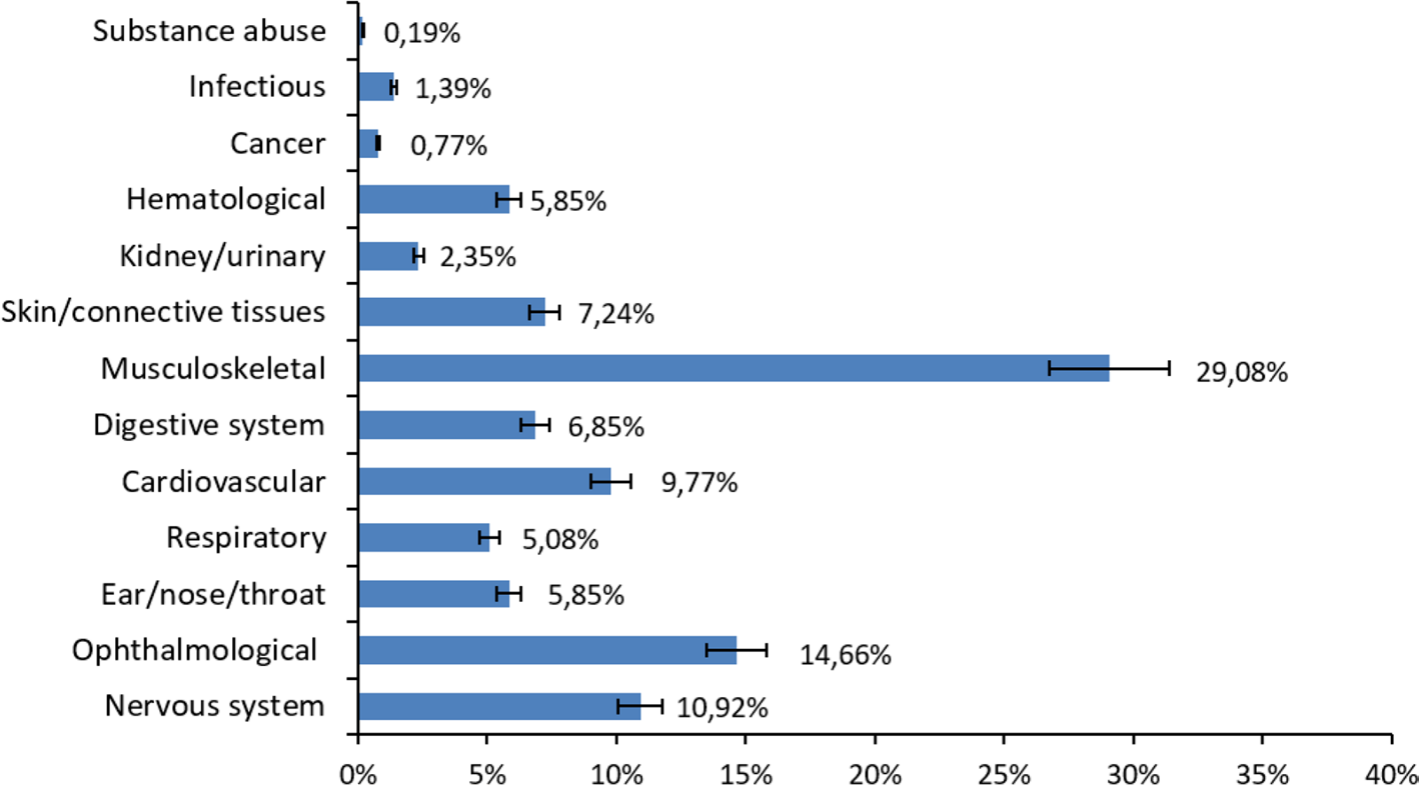
Medical disorders in employees using VDTs (*n* = 516). Variation error is 10%

Of the 2453 employees evaluated clinically, 1942 had no medical disorder, that is, they were able to perform their work optimally. In the remaining 511 employees, musculoskeletal and ophthalmological problems between them accounted for almost half (43.74%) of all conditions: 29.08% musculoskeletal problems and 14.66% ophthalmological problems (Fig. 1). Next in order of importance were diseases of the nervous system (10.92%), mainly headaches, nausea and stress, which also co-occurred frequently with musculoskeletal problems, corroborating the findings of other studies [13, 14]. Also important, although to a lesser extent, were diseases of the cardiovascular system (9.97%) (Table 1).

**Table 1:** Clinical variables for administrative VDT users according to the most probable medical disorders

Clinical variables	No disorder (*n* = 1942	Disorder			
		Musculoskeletal (*n* = 149)	Ophthalmological (*n* = 75)	Nervous (*n* = 56)	Cardiovascular (*n* = 50)
Sex	64.43%—men	67.11%—men	62.86%—men	55.36%—men	81.40%—men
	35.57%—women	32.89%—women	37.14%—women	44.64%—women	18.60%—women
	17.24 (< 0.001)	2.91 (0.004)	1.28 (0.2)	0.04 (0.97)	3.7 (< 0.001)
Age^a^	43.60% < 39 years	28.19% < 39 years	33.29% < 39 years	34.29% < 39 years	32.14% < 39 years
	56.40% ≥ 39 years	71.81% ≥ 39 years	66.71% ≥ 39 years	65.53% ≥ 39 years	67.86% ≥ 39 years
	25.66 (< 0.001)	6.96 (< 0.001)	3.96 (< 0.001)	3.25 (0.001)	3.38 (< 0.001)
BMI	50.72% < normal	44.97% < normal	54.29% < normal	46.43% < normal	37.21% < normal
	49.28% ≥ overweight	55.03% ≥ overweight	45.71% ≥ overweight	53.57% ≥ overweight	62.79% ≥ overweight
	9.62 (< 0.001)	2.71 (0.007)	0.24 (0.81)	1.4 (0.16)	2.65 (0.008)
Sleep quality	83.27%—good	79.19%—good	77.14%—good	67.64%—good	83.72%—good
	16.73%—variable	20.81%—variable	22.86%—variable	32.36%—variable	16.28%—variable
	53.64 (< 0.001)	5.97 (< 0.001)	3.81 (< 0.001)	1.83 (0.07)	4.04 (< 0.001)
Activity level	68.42%—yes	39.64%—yes	71.43%—yes	52.38%—yes	36.59%—yes
	34.58%—no	60.36%—no	28.57%—no	47.62%—no	63.41%—no
	24.68 (< 0.001)	4.06 (< 0.001)	2.79 (0.005)	0.5 (0.62)	2.74 (0.006)
Smoker^b^	16.68—yes	15.44—yes	20.02—yes	21.43—yes	23.26—yes
	83.32—no	84.56—no	79.98—no	78.57—no	76.74—no
	75.28 (< 0.001)	10.19 (< 0.001)	6.31 (< 0.001)	5.21 (< 0.001)	4.66 (< 0.001)
Alcohol user^c^	67.76—yes	63.09—yes	68.57—yes	69.64—yes	69.81—yes
	32.24—no	36.91—no	31.43—no	30.36—no	30.19—no
	23.39 (< 0.001)	1.89 (0.06)	2.29 (0.02)	2.13 (0.033)	2.04 (0.04)

^a^The age cutoff used was the mean (39 years)

^b^Both frequent and sporadic smokers

^c^Both habitual and sporadic/weekend consumers of alcohol

### Inference findings

**Fig. 2:**
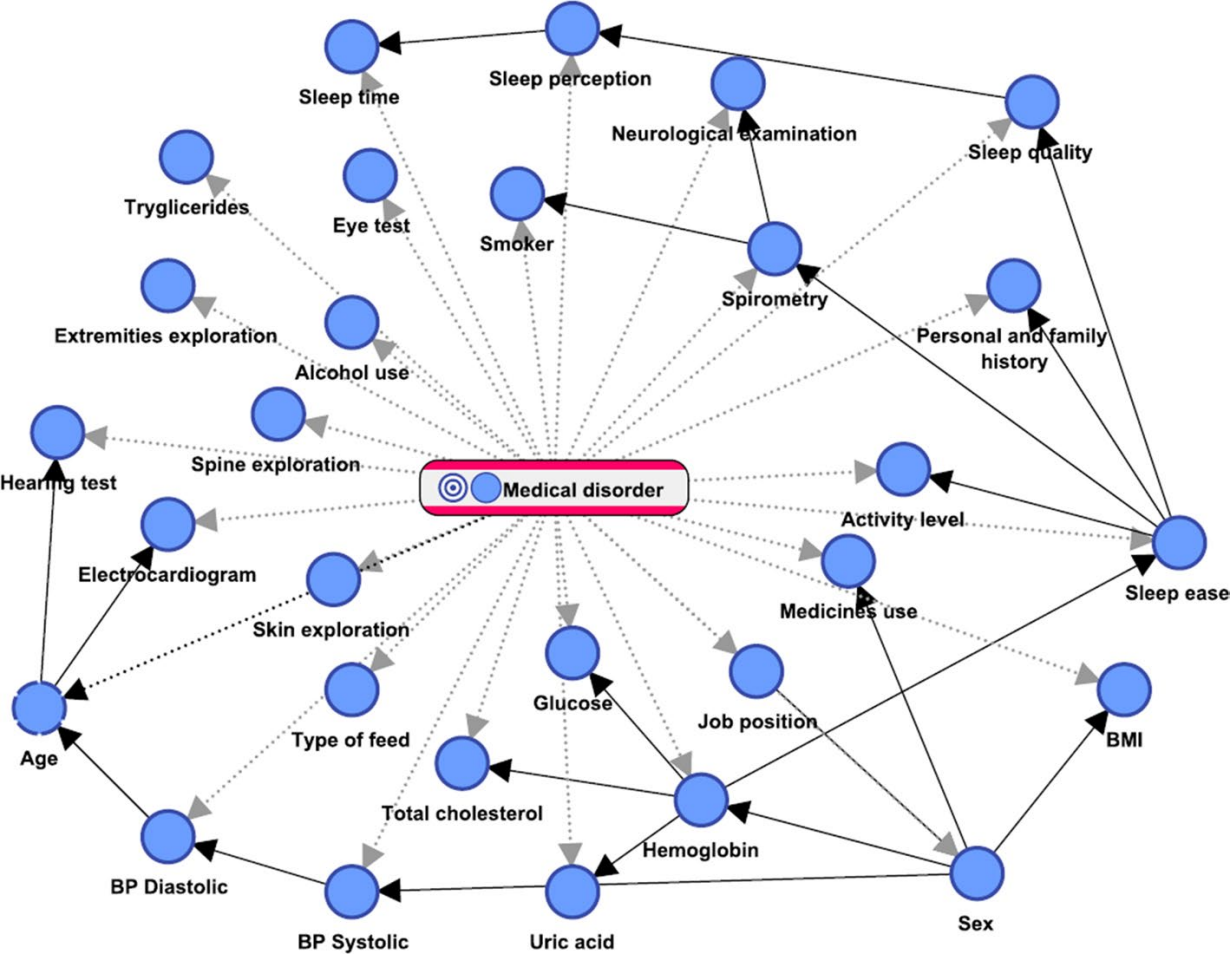
Supervised network built with the augmented Naive Bayes algorithm

By applying the Augmented Naive Bayes algorithm, the supervised network shown in Fig. 2 was built. This network allows inferring the Bayesian inference results included in Table 1, which shows the inferred states for the clinical variables for VDT users for the four most frequent disorders encountered in our sample (Fig. 1). The variables that contributed most information to the inference process were sex, age, BMI and sleep quality. Proportions were statistically tested to identify the clinical variables that were causally and significantly related to those disorders (shown in Table 1 together with the corresponding *p* value in parentheses).

The results indicate that age and smoking habits were significantly (*p* = 0.05) related to all the diseases. Most employees with musculoskeletal diseases (71.81%) were older than the average of 39 years, versus just over half of the healthy employees (57.62%). The same trend was also evident for ophthalmological and nervous system problems, although less emphatically: 66.71% and 65.53%, respectively, were older than the average age.

In relation to diseases of the nervous system, a key variable was sleep quality: 32.36% of employees with these conditions compared to 16.62% of healthy employees had variable sleep quality. As for employees with cardiovascular problems, the associated clinical variables were a BMI in the overweight category and low physical activity, as well as—to a lesser degree—smoking.

It was also found that sex was highly predictive of diseases of the cardiovascular and nervous systems, specifically, cardiovascular diseases for men and nervous disorders for women. For instance, while men represented 64.56% of healthy employees overall, the percentages with diseases of the cardiovascular and nervous system were 81.40% and 55.36%, respectively. Such marked differences were not evident for other diseases.

### Protocol strategies and cost utilities

**Fig. 3:**
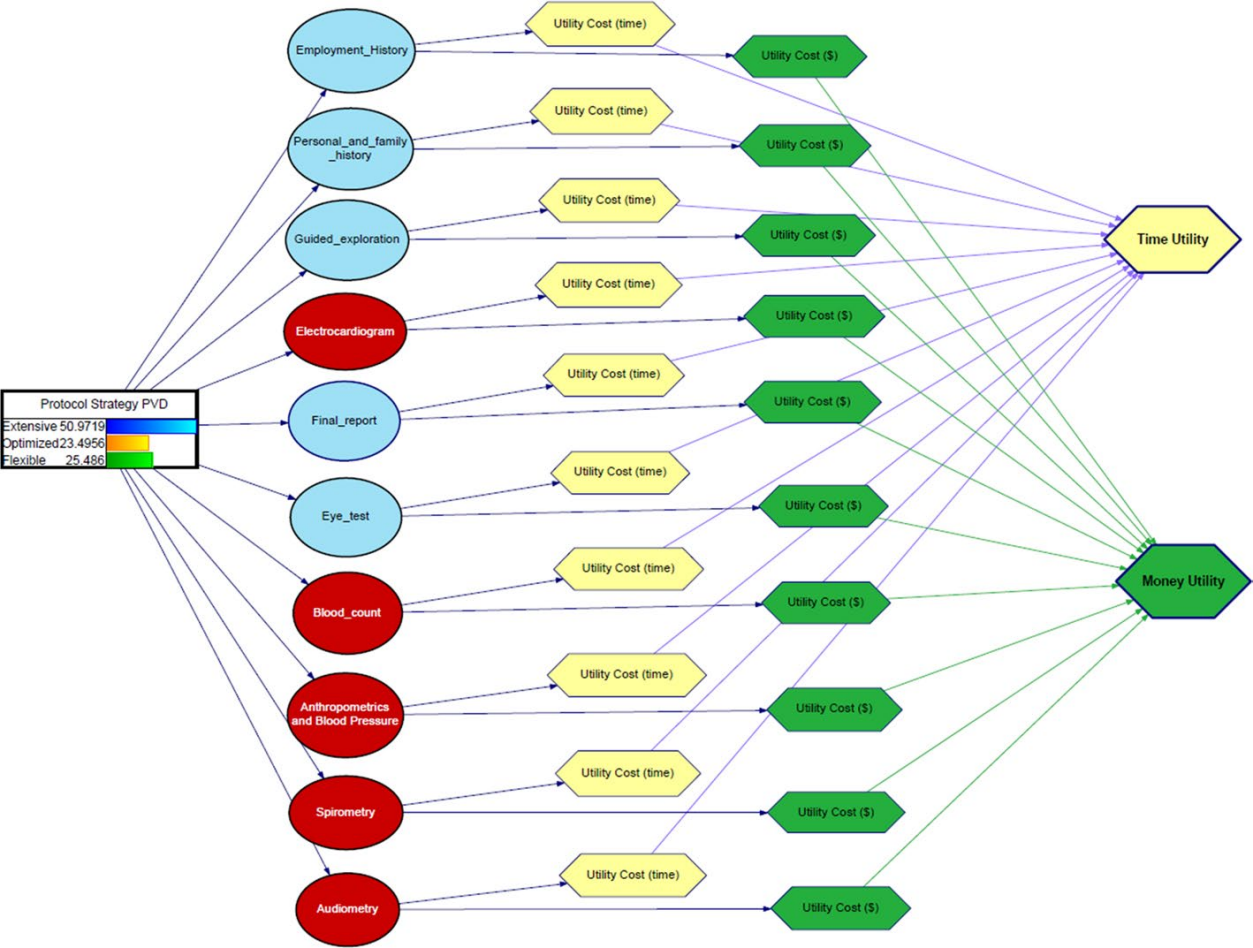
Influence diagram for the VDT protocol

Figure 3 depicts the influence diagram constructed to evaluate the extensive, optimized and flexible protocols. Table 2 summarizes six protocol strategies and their costs, one extensive (*A*), one optimized (*F*) and four flexible (*B*–*E*).

**Table 2:** Six protocol strategies and the associated costs

Protocol test	Protocol strategy					
	Extensive	Flexible				Optimized
	*A*	*B*	*C*	*D*	*E*	*F*
Employment history	✓	✓	✓	✓	✓	✓
Personal and family history	✓	✓	✓	✓	✓	✓
Guided exploration	✓	✓	✓	✓	✓	✓
Electrocardiogram	✓	✓	✓	✓	✓	
Final report	✓	✓	✓	✓	✓	✓
Eye test	✓	✓	✓	✓	✓	✓
Blood count	✓					
Anthropometrics and blood pressure	✓	✓	✓	✓		
Spirometry	✓	✓	✓			
Audiometry	✓	✓				
Time utility (min)	35	31	27	24	20.5	17
Money utility (USD)	16.7309	11.4933	10.2675	9.3481	8.2755	6.8043
Total utility (USD)	51.7309	32.4933	37.2675	33.3481	28.7755	23.8043
Total cost reduction		11%	23%	31%	41%	54%

The extensive strategy, currently used by the prevention service, includes all the medical tests, at a total utility cost of 51.7309 USD per employee. The additive linear utility nodes indicate that the time cost is 35 min, while the monetary cost is 16.7309 USD.

At the opposite extreme is the optimized strategy, consisting of five diagnostic tests directly related to the most probable diseases among VDT users. The optimized strategy reflects the minimum necessary set of medical tests necessary to guarantee the suitability of the employee to carry out their work with VDTs, as long as there are no particular health risks in their workplace that must be evaluated clinically. In this case, the total utility cost is 23.8043 USD, for a time cost of 17 min and a monetary cost of 6.8043 USD.

The flexible strategy is an approach based on performing diagnostic tests in accordance with clinical risk as assessed by medical staff. This strategy highlights the importance of a medical protocol that accurately reflects the real health risks to which an employee is exposed. For example, strategy B includes all diagnostic tests except the blood test, which in most cases should correspond to general medicine, and strategy C also excludes audiometry, which is appropriate in the case of employees not subject to sound thresholds higher than established by law [19]. The total cost will always lie somewhere between the cost of extensive and the optimized strategies.

Finally, the last row in Table 2 shows the percentage reduction in the total cost of each strategy with respect to the extensive strategy (*A*). The optimized strategy (*F*) reduces costs by more than half (54%), whereas the various flexible strategy options reduce costs in an interval that ranges from 11 (B) to 41% (*E*), depending on the diagnostic tests included on the basis of a risk assessment by medical staff.

## Discussion

**Fig. 4:**
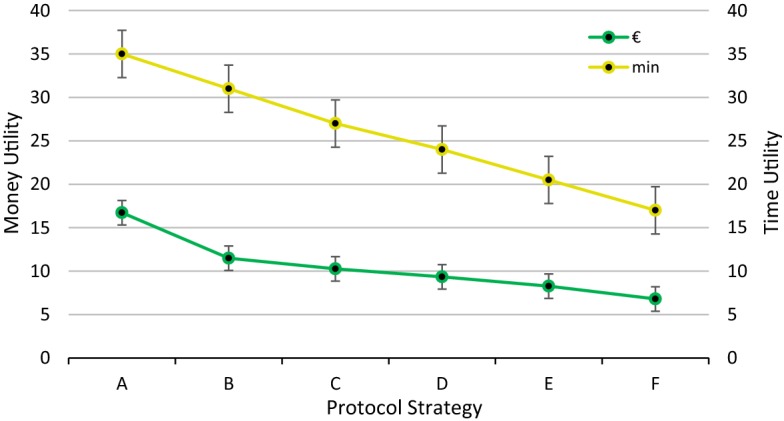
Monetary and time utility for each protocol strategy

Health surveillance has been contributing in recent times to duplicity in diagnostic testing and in the associated costs. In our research into occupational protocol strategies for employees using VDTs, affected mainly by musculoskeletal and ophthalmological disorders [9, 13, 14], we explored alternative strategies to the current extensive strategy of including a wide range of diagnostic tests. This extensive strategy is twice as inefficient in terms of time and monetary costs as an optimized strategy, which includes only diagnostic tests that focus exclusively on identifying disorders associated with real workplace risks (Fig. 4).

Between these two extremes (*A* and *F* in Fig. 4), we analysed the impact of the exclusion of specific diagnostic tests. While the reduction in the time cost was practically proportional for each of the flexible strategies (*B*–*E*), the reduction in the monetary cost was more varied. The main impact on monetary cost was exclusion of the blood count, a costly test compared to other tests (Table 3). On the other hand, the fact that other tests are relatively inexpensive raises the question as to whether their exclusion is practical from an employee health perspective.

**Table 3:** Diagnostic tests for administrative users of VDTs

Area	Specialist	Time cost per employee (min)	Monetary cost per employee (USD)
Employment history	Doctor	2.5	1.0509
Personal and family history	Doctor	3	1.2610
Guided exploration	Doctor	4	1.6814
Electrocardiogram	Doctor	3.5	1.4712
Final report	Doctor	4.5	1.8916
Eye test	Nurse	3	0.9194
Blood count	Nurse	4	5.2376
Anthropometrics and blood pressure	Nurse	3.5	1.0726
Spirometry	Nurse	3	0.9194
Audiometry	Nurse	4	1.2258

Cost was calculated according to the time spent on each test by the health specialist in proportion to their salary. Salaries are those indicated in the Spanish First National Collective Agreement for External Prevention Services [22]

### The benefits of flexibility

For employees who work at heights or who are exposed to chemical agents, a blood test clearly needs to be an essential component of health surveillance. However, this is not the case for employees using VDTs for administrative tasks. This difference in risk led us to propose four flexible strategies, customized by medical staff in terms of specific diagnostic testing according to workplace risk. A key element in ensuring the success of a flexible approach is that medical staff are informed regarding the most probable disorders in any particular group of employees—in our case, VDT users (Table 1). However, this kind of information would obviously need to be recorded over time to ensure optimally effective health surveillance [20].

Default strategies do not tend to represent the maximum long-term benefit, as they result in error and inefficacy. It is therefore important for protocols to adapt to changing circumstances. A protocol strategy in which the medical staff could decide what tests to perform in each case, while aware of their associated costs, would optimize diagnostic effectiveness. This kind of flexibility would also enable the detection of medical conditions that, while not directly associated with occupational risk, could cause the employee to leave their job. While this approach offers great potential, it would also require suitable planning of the medical agenda. In any case, treatment of detected disorders would always be the responsibility of the public health system or the employee’s private medical insurance.

### Limitations

The exclusion of certain medical tests in order to ensure a flexible or optimized protocol is a sensitive issue, as it may undermine employee trust in the quality of medical care and employer faith in health surveillance as a means to ensure the health of their staff. Occupational health surveillance has traditionally been associated only with broad-based medical examinations, with little attention paid to protocolization and its potential benefits. The outcome has been the performance of unnecessary diagnostic tests, merely out of habit, not because they are necessary to establish an employee’s capacity to do their job.

Protocolization has begun to receive attention as a consequence of greater awareness of health system costs and burdens. Although employees subjectively perceive occupational health surveillance to be better if they undergo a greater number of tests, our research shows that not all tests are equally significant. Thus, it is a viable approach to optimizing resource use and occupational health surveillance efficiency.

The need to raise awareness of the benefits of adopting this kind of strategy and to differentiate health care (public or private) from occupational health surveillance is unquestionable [21]. Our results for administrative users of VDTs demonstrate the importance of convincing employees and employers of the need for a more flexible approach to medical protocols.

## Conclusions

Many diagnostic tests included in occupational health surveillance protocols for administrative users of VDTs are unnecessary in terms of determining the employee’s capacity to perform their job. Different strategies can be considered, in accordance with the flexibility afforded to the medical team, regarding which diagnostic tests to perform based on the particular occupational risks to which an employee is exposed. We defined an optimized protocol based on the identification of disorders typically associated with the risks to which administrative VDT users are exposed, finding that medical protocols should ideally focus on specific musculoskeletal and ophthalmological disorders, also frequently associated with nervous system disorders. Our Bayesian inference procedure identified factors such as age, sleep quality, activity level, BMI and tobacco and alcohol use to be especially important in identifying the most common disorders affecting this group of employees. We reported overall savings of 11% to 41% from using a flexible approach to protocolization for administrative VDT users.

In conclusion, there is undoubtedly a need for education regarding the difference between general medical care and occupational health surveillance, as cultural and commercial biases affect effective decision-making within health systems. Our conclusions regarding VDT users need to be further explored in relation to the health surveillance of other occupational categories.

## Methods

### Sample and protocol description

We analysed 2453 health surveillance examinations of administrative employees (employed by Spanish companies mainly in the hospitality, medicine, restaurant, heritage and mining sectors), whose work involves use of VDTs. All medical examinations were carried out in Spain in the 7-year period January 2010 to December 2016 by authorized medical staff attached to the prevention service responsible for occupational health surveillance. In cooperation with the prevention service, the identity of the patients was coded to ensure their anonymity.

All the employees were medically examined according to the prevention service protocol for each medical area. Table 3 shows details of the medical areas, the medical staff who performed each test and the time and monetary cost per employee.

The protocolized health surveillance procedure is based on a team composed of an occupational medicine specialist and a specialist company nurse. While the nurse is qualified to perform tests such as analytical tests and spirometry, reviews of medical histories and guided explorations are of necessity performed by the doctor, who also concludes the report by taking the final decision on the employee’s health. The remaining tests are divided between the two professionals in such a way as to efficiently manage the medical agenda.

A medical examination requires around 35 min (divided equally between the doctor and the nurse) and costs 16.7308 USD. Each medical team was estimated as being able to perform a maximum of 15 medical examinations daily. Health surveillance was structured in terms of collective surveillance based on documentation (company schedules, workplace protocols, reports with epidemiological studies), and individual surveillance based on preventive medical examinations. Since individual monitoring is the area where medical decision-making can potentially be most optimized, we statistically evaluated the implications for the employee’s health of performing and not performing certain medical tests.

### Statistical analysis

Based on the data obtained from the medical examinations, a Bayesian network was machine-learned in order to create a supervised structure [23] where the prediction objective (target) was the employee’s disorder. We thus obtained a model of the data that allowed us to infer the main issues affecting the health of employees using VDTs.

A Bayesian network is defined as a triplet (*X*, *G*, *P*), where *X* = (*X*_1_,*X*_2_,…,*X*_*n*_) represents clinical variables and possible diseases, where G is a directed acyclic graph (DAG) composed of nodes labelled with elements of *X* and arcs indicating an influence or causal relationship between nodes, and where *P* is a joint probability distribution on *X*, so that each clinical variable and each disease is assigned a table of conditional probabilities that define the probability of different states.

Bayesian networks assume, according to the decomposition theorem, that a node depends only on its parents, with a parent node understood to be a node from which there is a descendent via an arc of the graph *G* [24]. In other words, the parent node is the causal node and a child node is the effect node. Consequently, to specify an (*X*, *G*, *P*) Bayesian network, given the parents, a conditional probability distribution is necessary for each factor. Note that using machine learning algorithms to perform structural learning of graph *G* and to calculate the joint probability distribution *P*, we avoid having to manually assign dependency relationships between variables—a subjective procedure open to error, especially when numerous variables are involved.

Of the structural learning algorithms available nowadays we used Augmented Naive Bayes, whose architecture is based on a naive structure enriched by the relationships between the different nodes other than the target node (the common parent) [25]. Although other algorithms such as Tree Augmented Naive Bayes and Markov Blanket were simulated, they did not improve the predictions of the network. The training phase of the network was carried out from the dataset. That is to say, the probabilities tables associated with each node are calculated according to the structure and information provided in the database. The method implemented is the so-called maximum likelihood estimator, where probabilities are estimated based on the frequencies of the data.

Bayesian networks can be used for reasoning in either direction: for predictive reasoning, from causal nodes (clinical variables) to effect nodes (diseases) following the direction of the connecting arcs, or in the reverse direction, for diagnostic reasoning, from effect nodes (diseases) to causal nodes (clinical variables). Intercausal reasoning is another possible kind of inference that examines the relationship between causal nodes that share the same effect node.

Bayesian networks are graphic tools that, unlike other statistical methods, intuitively represent the cause–effect relationships between the factors. By applying them to health surveillance databases, they can offer a very realistic view of the causes of occupational diseases. Bayesian networks also allow sensitivity analysis by quantifying the impact that a small change in one factor produces on other factors.

Bayesian statistics have amply demonstrated their usefulness in the medical field and in decision-making in general [26]. The Bayesian procedure was implemented using BayesiaLab software, version 7.0.1 (2018) [27].

### Protocol strategies

An influence diagram was created to explore the options available in configuring the most appropriate medical protocol and to compute the associated costs. The decision node represented three possible protocol strategies.

- Extensive: this represents the current situation, in which all protocol diagnostic tests are performed by default.
- Optimized: this represents maximum cost reduction, whereby the only diagnostic tests performed are those that can potentially detect occupational risk-related problems in employees.
- Flexible: this represents an intermediate scenario, in which medical staff decide what tests to perform based on the history and clinical picture of the particular employee.

The strategy for each protocol is based on the performance of specific medical tests. Each test is represented in the influence diagram by a probabilistic node, with each such node having two utility nodes that evaluate the time and monetary costs associated with each test (see Table 3). Finally, by incorporating two additive linear utility nodes in the diagram, we can independently compute total utility from the time and monetary cost utility nodes.

Influence diagrams are, mathematically speaking, a generalization of a Bayesian network. They are a compact, intuitive tool for dealing with decisions such as those described here and have already been used successfully in the analysis of medical decisions. The decision modelling software used for this task was GeNIe version 2.2 (2018) [28].

## Data Availability

Not applicable.

## Abbreviations

VDTs: video display terminals
DAG: directed acyclic graph
BMI: body mass index
